# Colorectal cancer risk variants rs10161980 and rs7495132 are associated with cancer survival outcome by a recessive mode of inheritance

**DOI:** 10.1002/ijc.33465

**Published:** 2021-01-23

**Authors:** Yazhou He, Maria Timofeeva, Xiaomeng Zhang, Wei Xu, Xue Li, Farhat V. N. Din, Victoria Svinti, Susan M. Farrington, Harry Campbell, Malcolm G. Dunlop, Evropi Theodoratou

**Affiliations:** ^1^ Department of Oncology, West China School of Public Health and West China Fourth Hospital Sichuan University Chengdu People's Republic of China; ^2^ Cancer Research UK Edinburgh Centre, Medical Research Council Institute of Genetics & Molecular Medicine, Western General Hospital The University of Edinburgh Edinburgh UK; ^3^ Colon Cancer Genetics Group, Medical Research Council Human Genetics Unit, Medical Research Council Institute of Genetics & Molecular Medicine, Western General Hospital The University of Edinburgh Edinburgh UK; ^4^ Centre for Global Health, Usher Institute The University of Edinburgh Edinburgh UK; ^5^ Danish Institute for Advanced Study (DIAS), Department of Public Health University of Southern Denmark Odense Denmark; ^6^ School of Public Health Zhejiang University Hangzhou People's Republic of China

**Keywords:** colorectal cancer, germline genetic variants, recessive model, survival outcomes

## Abstract

Previous studies using additive genetic models failed to identify robust evidence of associations between colorectal cancer (CRC) risk variants and survival outcomes. However, additive models can be prone to false negative detection if the underlying inheritance mode is recessive. Here, we tested all currently known CRC‐risk variants (n = 129) in a discovery analysis of 5675 patients from a Scottish cohort. Significant associations were then validated in 2474 CRC cases from UK Biobank. We found that the TT genotype of the intron variant rs7495132 in the *CRTC3* gene was associated with clinically relevant poorer CRC‐specific survival in both the discovery (hazard ratio [HR] = 1.97, 95% confidence interval [CI] = 1.41‐2.74, *P* = 6.1 × 10^−5^) and validation analysis (HR = 1.69, 95% CI = 1.03‐2.79, *P* = .038). In addition, the GG genotype of rs10161980 (intronic variant of *AL139383.1* lncRNA) was associated with worse overall survival in the discovery cohort (HR = 1.24, 95% CI = 1.10‐1.39, *P* = 3.4 × 10^−4^) and CRC‐specific survival in the validation cohort (HR = 1.26, 95% CI = 1.01‐1.56, *P* = .040). Our findings show that common genetic risk factors can also influence CRC survival outcome.

**Abbreviations**
AJCC: American Joint Committee on Cancer
CI: confidence interval
CRC: colorectal cancer
GWAS: genome‐wide association study
HR: hazard ratio
SOCCS: Study of Colorectal Cancer in Scotland

**What's new?**

To date, there is a dearth of well‐powered studies examining the effect of common germline variants associated with colorectal cancer risk on subsequent survival outcomes. While studies using additive genetic models have failed to reveal robust associations between colorectal cancer risk variants and survival outcomes, additive models can be prone to false‐negative detection if the underlying inheritance mode is recessive. Here, the authors identified two genetic variants with significant recessive effects on survival outcomes in colorectal cancer. Altogether, the findings provide novel insight into the genetic architecture influencing post‐diagnosis survival in colorectal cancer patients.

## 1 INTRODUCTION

Previous evidence have shown a familial contribution to survival outcomes of colorectal cancer (CRC), indicating that heritable germline genetic components can contribute to survival outcome.^1^ Statistical association between germline variation and survival should be free from confounding and reverse causation, and so could have utility in *prediction* of cancer outcomes. Genetic variants involved in CRC pathogenesis may also influence subsequent survival outcomes in affected carriers. However, previous studies of low penetrance common genetic variance found inconsistent results using additive genetic models. Hence, no robust associations have been established between any common germline variation and CRC survival outcome.^2^, ^3^ Additive models assume a linear relationship between risk and each allele and is widely used in genome‐wide association studies (GWAS).^4^ However, simulation studies show that the additive approach has low statistical power to identify effects of variants with true mode of inheritance being recessive.^4^ We therefore investigated potential recessive genetic effects of variants known to be associated with CRC *risk*, in order to determine whether such variants also had recessive effects on survival outcomes.

## 2 MATERIALS AND METHODS

We tested associations between survival outcomes of CRC patients and a total of 129 known genetic variants (linkage disequilibrium *r* ^2^ < 0.2) associated with CRC risk reported by two recent GWAS analyses^4^, ^5^ (details summarised in Supplementary Table S1). Technical details of genotyping, imputation and quality control steps can be found in previous publications.^6^

We studied 5675 CRC patients with GWAS data from the Study of Colorectal Cancer in Scotland (SOCCS) (discovery set). Age at diagnosis, sex and tumour stage were collected as covariates. Statistical power of discovery analysis was estimated for variants with varied genotype frequencies and effect sizes using the method proposed by Freedman^7^ (Type I error rate = 0.0005). The Cox hazards model was employed to estimate hazard ratios (HRs) along with 95% confidence intervals (CIs) of homozygotes of minor alleles on overall and CRC‐specific survival. We used the false positive rates (FDR) approach to correct for multiple testing (*P* < .05 was the significance threshold after correction). We checked the proportional hazards assumption of the Cox model for each identified variant by testing the association between the Schoenfeld residuals and follow‐up time using the SOCCS cohort.^8^ If time‐dependent trends of Schoenfeld residuals were detected (*P* < .05), which indicated potential violation of the proportional hazards assumption, we fitted a parametric Weibull model to estimate the genetic effects on survival outcomes.^9^ We also explored the associations between the 129 included variants and survival outcomes stratified by sex.

Significant associations identified from the SOCCS were then validated in an independent population‐based prospective cohort composed of 2474 incident CRC cases from the UK Biobank. Only age at diagnosis and sex were adjusted in the model due to CRC stage being unavailable in the UK Biobank. Given different covariates used, meta‐analysis combining the two cohorts was not performed. Ethics approval of the study cohorts is presented elsewhere.^10^ The diagram of patient selection for the two study cohorts is presented in Supplementary Figure S1.

## 3 RESULTS AND DISCUSSION

Patient characteristics for the study cohorts are presented in Table 1. Given 5675 included cases, the discovery analysis yielded 88% (overall survival) and 66% (CRC‐specific survival) power for 82 of the 129 (64%) included variants (homozygous genotype frequency >0.05) to detect a HR of at least 1.50. Power estimates of variants with varied genotype frequencies and effect sizes are presented in Supplementary Table S2.

**TABLE 1:** Summarised characteristics of the discovery and validation cohorts

Variables^a^	SOCCS (n = 5675)	UK Biobank (n = 2474)
Age at diagnosis (years)	64.5 (54.6‐71.6)	64.9 (61.2‐69.6)
Sex		
Male	3235 (57%)	1035 (42%)
Female	2440 (43%)	1439 (58%)
AJCC stage		
I	1005 (17.7%)	NA
II	1891 (33.3%)	
III	1995 (35.2%)	
IV	784 (13.8%)	

Abbreviations: AJCC, the American Joint Committee on Cancer; NA, not available.

^a^ Continuous variables are presented with median and interquartile range.

**FIGURE 1:**
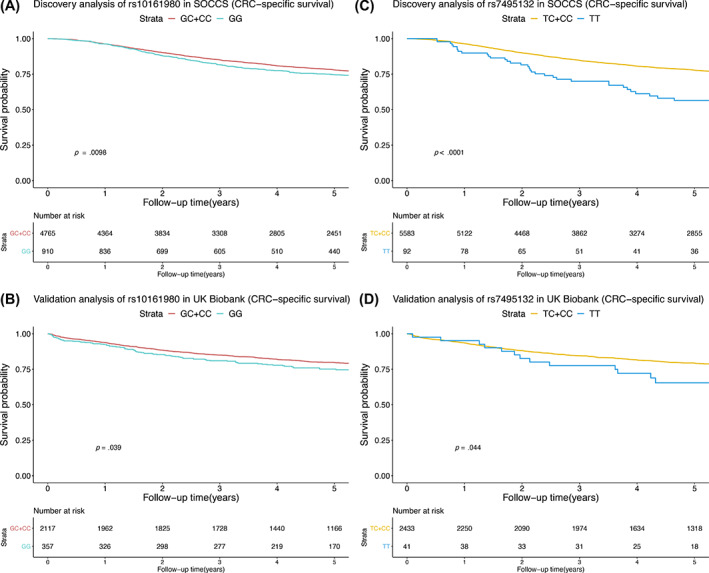
Kaplan‐Meier survival estimates of colorectal cancer (CRC)‐specific survival stratified by rs10161980 and rs7495132 (A, rs10161980 in SOCCS; B, rs10161980 in UK Biobank; C, rs7495132 in SOCCS; D, rs7495132 in UK Biobank) [Color figure can be viewed at wileyonlinelibrary.com]

In the discovery analysis, we found 12 variants associated with overall survival and 9 variants associated with CRC‐specific survival at nominal significance (uncorrected *P* < .05; Table 2). After FDR correction, we identified that the GG genotype of the variant rs10161980 was associated with clinically relevant inferior overall survival (HR = 1.24, 95% CI = 1.10‐1.39, uncorrected *P* = 3.4 × 10^−4^, FDR corrected *P* = .022). However, this effect was not significant on CRC‐specific survival after FDR correction (HR = 1.30, 95% CI = 1.11‐1.52, uncorrected *P* = .005, FDR corrected *P* = .196). In addition to rs10161980, the TT genotype of the variant rs7495132 was significantly associated with worse CRC‐specific survival (HR = 1.97, 95% CI = 1.41‐2.74, uncorrected *P* = 6.1 × 10^−5^, FDR corrected *P* = .008), whereas the association with overall survival did not survive the FDR correction (HR = 1.40, 95% CI = 1.01‐1.93, uncorrected *P* = .045, FDR corrected *P* = .467). No significant deviation from the proportional hazards assumption was observed for either variants (*P* > .05). We then validated the two observed signals using the UK Biobank cohort (N = 2474). The GG genotype of rs10161980 was significantly associated with worse CRC‐specific survival (HR = 1.26, 95% CI = 1.01‐1.56, *P* = .040), but not significantly associated with overall survival (HR = 1.21, 95% CI = 0.99‐1.46, *P* = .057). As for the other variant rs7495132, we replicated the observed association between the TT genotype and CRC‐specific survival in the UK Biobank (HR = 1.69, 95% CI = 1.03‐2.79, *P* = .038), although no significant effect was detected of this variant on overall survival (HR = 1.39, 95% CI = 0.86‐2.25, *P* = .179). Kaplan‐Meier survival estimates of these two variants in both discovery and validation analysis are plotted in Figure 1 (CRC‐specific survival) and Supplementary Figure S2 for overall survival.

**TABLE 2:** Summary of associations (*P* < .05) between colorectal cancer (CRC)‐risk variants and survival outcomes of CRC patients in the Study of Colorectal Cancer in Scotland (SOCCS) study under a recessive model

Variant	MA	RGF	HR (95%CI)	*P* (uncorrected)	Pfdr
OS					
**rs10161980**	**G**	**0.12**	**1.24 (1.10‐1.39)**	**3.40E‐04**	**.022**
rs174537	T	0.09	1.23 (1.07‐1.41)	.003	.091
rs6066825	G	0.24	1.22 (1.07‐1.40)	.003	.091
rs73975588	C	0.01	0.55 (0.35‐0.86)	.009	.235
rs3087967	C	0.07	0.89 (0.82‐0.98)	.014	.311
rs3217810	T	0.003	1.42 (1.06‐1.92)	.021	.380
rs78341008	C	0.001	1.79 (1.08‐2.98)	.024	.395
rs35509282	A	0.05	0.61 (0.40‐0.96)	.030	.437
rs10951878	T	0.20	0.89 (0.80‐0.99)	.035	.454
rs35360328	A	0.01	1.35 (1.01‐1.80)	.041	.467
rs7495132	T	0.02	1.40 (1.01‐1.93)	.045	.467
rs16892766	C	0.01	0.55 (0.30‐1.00)	.049	.467
CSS					
**rs7495132**	**T**	**0.02**	**1.97 (1.41‐2.74)**	**6.10E‐05**	**.008**
rs6066825	G	0.24	1.30 (1.11‐1.52)	.001	.063
rs10161980	G	0.12	1.22 (1.06‐1.40)	.005	.196
rs9537521	A	0.03	1.22 (1.06‐1.41)	.006	.201
rs4811050	A	0.04	1.38 (1.08‐1.78)	.012	.301
rs35509282	A	0.05	0.52 (0.30‐0.90)	.019	.414
rs10951878	T	0.20	0.87 (0.76‐0.99)	.030	.492
rs3217810	T	0.003	1.47 (1.04‐2.09)	.030	.492
rs13020391	T	0.09	1.17 (1.01‐1.35)	.037	.536

*Note*: Bold values indicate significant associations after FDR correction.

Abbreviations: CI, confidence interval; CSS, CRC‐specific survival; HR, hazard ratio; MA, minor allele; OS, overall survival; Pfdr, *P*‐values adjusted using the false positive rate approach; RGF, rare genotype frequency.

With regard to stratified analysis by sex, the variant rs7495132 remained significantly associated with CRC‐specific survival of male patients (TT genotype: HR = 2.35, 95% CI = 1.54‐3.60, uncorrected *P* = 8.0 × 10^−5^, FDR corrected *P* = .010), whereas rs10161980 was observed to be associated with overall survival of female patients from the SOCCS cohort (GG genotype: HR = 1.38, 95% CI = 1.15‐1.65, uncorrected *P* = .001, FDR corrected *P* = .041). In addition, we identified significant association between the TT genotype of the variant rs174537 and worse overall survival among female patients from the SOCCS. However, we failed to validate any of these associations by conducting sex‐stratified analysis using the UK Biobank cohort (details in Supplementary Table S4).

In our study, we identified two CRC‐risk variants (rs7495132 and rs10161980) with significant effects on survival outcomes of CRC. Notably, both variants, although not reaching statistical significance, showed the same direction but smaller magnitude of effects in our previous analysis using additive model,^2^ indicating the underlying recessive model of inheritance for these two variants.

The T allele of rs7495132 was identified as a CRC‐risk increasing allele in the original GWAS meta‐analysis.^6^ Located in chromosome 15, rs7495132 is an intron variant of the *CRTC3* gene which encodes the protein CREB regulated transcription co‐activator 3 (CRTC3) and is associated with human T‐cell leukaemia virus infection. According to the results of Genotype‐Tissue Expression project (https://gtexportal.org/home/), the T allele of rs7495132 is associated with lower expression of the *CRTC3* gene in multiple human tissues such as musculoskeletal tissue and subcutaneous adipose tissue, although no significant association is present in colonic tissue. Previous evidence showed that the CRTC3 protein can regulate energy balance and is associated with weight gain in a mouse model.^11^ To explore the potential role of adiposity in the observed genetic association, we compared the BMI level among CRC patients of different rs7495132 genotypes using the UK Biobank cohort. However, no significant difference was detected (mean BMI level of TT vs TC+CC genotypes: 27.64 vs 27.97, *P* = .595). We also added BMI as a covariate in the survival model, and observed that the BMI level was not associated with survival outcomes (CRC‐specific survival: HR = 1.00, 95% CI = 0.98‐1.02, *P* = .938), whereas the genetic association between rs7495132 and survival outcomes remained significant in the multivariable model. These results could not rule out adiposity as a potential mediator in the observed genetic association. Future research is needed to further reveal the possible biological function of rs7495132 in the adiposity‐mediated pathway involved in CRC progression.

With respect to the other variant rs10161980, it is an intron variant of the *AL139383.1* LncRNA located in chromosome 13. Currently, little is known for potential biological implications of this gene, and there has been a paucity of evidence that rs10161980 can affect expression of any genes. Notably, the G allele of rs10161980 was identified as the CRC‐risk decreasing allele in the original GWAS.^6^ This indicates that the variant may be involved in different biological pathways of CRC carcinogenesis and tumour progression. It should be noted that a chance finding could not be confidently excluded despite the validation in our study. Therefore, future large population‐based studies are also needed to confirm this association.

Given the limited sample size of the study cohorts and the number of variants tested, our study was underpowered to detect small recessive genetic effects (HR < 1.20), especially for variants with low homozygous genotype frequencies (<0.30). Another limitation is that American Joint Committee on Cancer (AJCC) stage was not adjusted in replication analysis due to unavailable data. We re‐estimate the effects of these two variants in SOCCS adjusted for only age and sex, and the results remained statistically significant (Supplementary Table S3).

Our study identifies two CRC‐risk variants (rs10161980 and rs7495132) that have recessive genetic effects on survival outcomes of CRC. Currently AJCC stage is used as a blunt indicator of the need for adjuvant chemotherapy. The genetic associations discovered in our study provide prospects for future investigations to incorporate rs10161980 and rs7495132 genotypes into individual prediction models for survival outcomes. In addition, our findings improve the understanding of the genetic architecture for prognosis of CRC patients. Future research is warranted to illuminate biological mechanisms through which these two variants influence CRC progression. Our findings also suggest that recessive genetic effects should be considered in future genetic association studies on survival of CRC.

## CONFLICT OF INTEREST

All authors declared no potential conflicts of interest.

## Supporting information


**Appendix S1:** Supplementary Information

## Data Availability

The datasets used and/or analysed during the current study are available from the corresponding author on reasonable request. The data are not publicly available due to privacy or ethical restrictions.
