# Supplementary material for: Colorectal cancer risk variants rs10161980 and rs7495132 are associated with cancer survival outcome by a recessive mode of inheritance
Source: Int J Cancer. 2021 Jan 23;148(11):2774–8. doi: 10.1002/ijc.33465 (PMC8614120; doi:10.1002/ijc.33465)
Supplement: Supplementary file 1 — Appendix S1: Supplementary Information [file IJC-148-2774-s001.pdf]

Colorectal cancer risk variants rs10161980 and rs7495132 are associated with cancer survival outcome by a recessive mode of inheritance.

Authors: Yazhou He, Maria Timofeeva, Xiaomeng Zhang, Wei Xu, Xue Li, Farhat VN. Din, Victoria Svinti, Susan M. Farrington, Harry Campbell, Malcolm G. Dunlop, Evropi Theodoratou

Contents:

Supplementary Tables:

Table S1 Basic characteristics of 129 genetic variants associated with CRC risk

Table S2 Power estimates of different genotype frequencies and effect sizes

Table S3 Effect estimates of rs7495132 and rs10161980 in SOCCS adjusting for age and sex

Table S4 Summarised results of genetic associations (p<0.05 in discovery set) stratified by sex

Supplementary Figures:

Figure S1 Flow chart of patient selection for the two study cohorts

Figure S2 Kaplan-Meier survival estimates of overall survival stratified by rs10161980 and rs7495132 (A: rs10161980 in SOCCS, B: rs10161980 in UK Biobank; C: rs7495132 in SOCCS; D: rs7495132 in UK Biobank)

Table S1 Basic characteristics of 129 genetic variants associated with CRC risk

Variant	Locus	MA	MGF	Gene	Reference
rs12143541	1p32.3	G	0.024	*TTC22*	Law, 2019
rs61776719	1p34.3	C	0.288	*FHL3*	Law, 2019
rs72647484	1p36.12	T	0.010	*Intergenic*	Law, 2019
rs10911251	1q25.3	A	0.187	*LAMC1*	Law, 2019
rs6658977	1q41	T	0.131	*LINC02257*	Law, 2019
rs11692435	2q11.2	G	0.008	*ACTR1B*	Law, 2019
rs448513	2q24.2	C	0.109	*TANC1*	Huyghe, 2019
rs11903757	2q32.3	C	0.028	*Intergenic*	Law, 2019
rs11893063	2q33.1	A	0.209	*AC019330.1*	Law, 2019
rs7593422	2q33.1	T	0.312	*SATB2*	Law, 2019
rs13020391	2q35	C	0.146	*PNKD*	Law, 2019
rs2279290	3p14.1	G	0.030	*LRIG1*	Law, 2019
rs9831861	3p21.1	G	0.346	*AC096887.1*	Law, 2019
rs35360328	3p22.1	A	0.023	*Intergenic*	Law, 2019
rs12635946	3q13.2	C	0.124	*Intergenic*	Law, 2019
rs72942485	3q13.2	G	0.001	*BOC*	Huyghe, 2019
rs10049390	3q22.2	A	0.427	*SLCO2A1*	Huyghe, 2019
rs10936599	3q26.2	C	0.055	*MYNN*	Law, 2019
rs1370821	4q22.2	T	0.193	*Intergenic*	Law, 2019
rs1391441	4q24	A	0.495	*TET2*	Huyghe, 2019
rs17035289	4q24	T	0.019	*Intergenic*	Law, 2019
rs3987	4q26	G	0.123	*LINC02264*	Law, 2019
rs75686861	4q31.21	A	0.011	*HHIP*	Law, 2019
rs186722897	4q32.2	T	0.003	*Intergenic*	Law, 2019
rs35509282	4q32.2	A	0.014	*Intergenic*	Law, 2019
rs1445011	5p13.1	C	0.092	*Intergenic*	Law, 2019

rs7708610	5p13.1	A	0.132	*Intergenic*	Huyghe, 2019
rs2735940	5p15.33	A	0.334	*TERT*	Law, 2019
rs77776598	5p15.33	C	0.005	*SLC6A18*	Law, 2019
rs12522693	5q23.3	G	0.020	*Intergenic*	Law, 2019
rs639933	5q31.1	C	0.375	*C5orf66*	Law, 2019
rs647161	5q31.1	A	0.448	*C5orf66*	Law, 2019
rs62404966	6p12.1	C	0.058	*BMP5*	Law, 2019
rs4711689	6p21.1	A	0.356	*TFEB*	Law, 2019
rs6933790	6p21.1	T	0.024	*TFEB*	Law, 2019
rs1321310	6p21.2	C	0.064	*Intergenic*	Law, 2019
rs16878812	6p21.31	A	0.012	*FKBP5*	Law, 2019
rs2516420	6p21.32	C	0.055	*HCP5*	Huyghe, 2019
rs9271770	6p21.32	A	0.673	*HLA-DQA1*	Law, 2019
rs3131043	6p21.33	G	0.196	*HCG20*	Law, 2019
rs2070699	6p24.1	T	0.013	*EDN1*	Law, 2019
rs6928864	6q21	C	0.007	*Intergenic*	Law, 2019
rs10951878	7p12.3	C	0.249	*AC004870.4*	Law, 2019
rs3801081	7p12.3	G	0.453	*TNS3*	Law, 2019
rs12672022	7p13	T	0.028	*TBRG4*	Huyghe, 2019
rs16892766	8q23.3	C	0.008	*Intergenic*	Law, 2019
rs4313119	8q24.21	G	0.042	*Intergenic*	Huyghe, 2019
rs6983267	8q24.21	G	0.197	*CASC8*	Law, 2019
rs1412834	9p21.3	T	0.262	*CDKN2B-AS1*	Law, 2019
rs34405347	9q22.33	T	0.004	*Intergenic*	Huyghe, 2019
rs10980628	9q31.3	C	0.050	*LPAR1*	Huyghe, 2019
rs10795668	10p14	G	0.103	*RNA5SP299*	Law, 2019
rs10994860	10q11.23	C	0.032	*A1CF*	Law, 2019
rs704017	10q22.3	G	0.351	*ZMIZ1-AS1*	Law, 2019

rs1035209	10q24.2	T	0.041	*Intergenic*	Law, 2019
rs4919687	10q24.32	G	0.082	*CYP17A1*	Law, 2019
rs11196171	10q25.2	G	0.049	*TCF7L2*	Law, 2019
rs12241008	10q25.2	C	0.011	*VTI1A*	Law, 2019
rs4450168	11p15.4	C	0.030	*SBF2*	Law, 2019
rs174537	11q12.2	G	0.110	*MYRF*	Law, 2019
rs3824999	11q13.4	G	0.261	*POLD3*	Law, 2019
rs4944940	11q13.4	G	0.001	*CHRDL2*	Law, 2019
rs2186607	11q22.1	T	0.468	*TRPC6*	Huyghe, 2019
rs3087967	11q23.1	T	0.454	*C11orf53*	Law, 2019
rs2238126	12p13.2	G	0.252	*ETV6*	Law, 2019
rs10849432	12p13.31	T	0.198	*Intergenic*	Law, 2019
rs10849438	12p13.31	G	0.016	*Intergenic*	Law, 2019
rs11064437	12p13.31	C	0.000	*TPI1/RPL13P5*	Law, 2019
rs10774214	12p13.32	T	0.411	*CCND2-AS1*	Law, 2019
rs3217810	12p13.32	T	0.017	*CCND2*	Law, 2019
rs3217874	12p13.32	T	0.224	*CCND2*	Huyghe, 2019
rs11610543	12q12	G	0.229	*Intergenic*	Huyghe, 2019
rs11169552	12q13.13	C	0.066	*ATF1*	Law, 2019
rs4759277	12q13.3	A	0.141	*LRP1*	Huyghe, 2019
rs7398375	12q13.3	C	0.059	*LRP1*	Law, 2019
rs3184504	12q24.12	C	0.267	*SH2B3*	Law, 2019
rs72013726	12q24.21	C	0.290	*Intergenic*	Law, 2019
rs73208120	12q24.22	G	0.011	*NOS1*	Law, 2019
rs10161980	13q13.2	C	0.160	*AL139383.1*	Law, 2019
rs12427600	13q13.3	C	0.063	*SMAD9*	Law, 2019
rs45597035	13q22.1	A	0.107	*KLF5*	Law, 2019
rs78341008	13q22.1	C	0.006	*Intergenic*	Huyghe, 2019

rs1330889	13q22.3	C	0.227	*LINC00446*	Law, 2019
rs7993934	13q34	T	0.422	*COL4A2*	Law, 2019
rs1570405	14q22.2	G	0.455	*Intergenic*	Law, 2019
rs35107139	14q22.2	C	0.163	*BMP4*	Law, 2019
rs17094983	14q23.1	G	0.012	*LINC01500*	Huyghe, 2019
rs11632715	15q13.3	A	0.232	*Intergenic*	Law, 2019
rs16959063	15q13.3	A	0.000	*FMN1*	Law, 2019
rs16969681	15q13.3	T	0.010	*SCG5*	Law, 2019
rs17816465	15q13.3	A	0.045	*FMN1*	Law, 2019
rs73376930	15q13.3	G	0.048	*GREM1*	Law, 2019
rs4776316	15q22.31	A	0.062	*SMAD6*	Law, 2019
rs56324967	15q22.33	C	0.466	*SMAD3*	Huyghe, 2019
rs10152518	15q23	G	0.342	*Intergenic*	Law, 2019
rs7495132	15q26.1	T	0.016	*CRTC3*	Law, 2019
rs9929218	16q22.1	G	0.076	*CDH1*	Law, 2019
rs61336918	16q23.2	A	0.451	*Intergenic*	Law, 2019
rs2696839	16q24.1	G	0.004	*Intergenic*	Law, 2019
rs847208	16q24.1	A	0.399	*LINC01081*	Law, 2019
rs899244	16q24.1	T	0.049	*AC009154.1*	Law, 2019
rs1078643	17p12	A	0.410	*TMEM238L*	Law, 2019
rs12603526	17p13.3	C	0.000	*NXN*	Law, 2019
rs73975588	17p13.3	A	0.016	*NXN*	Law, 2019
rs17836917	17q12	G	0.000	*ASIC2*	Law, 2019
rs983318	17q24.3	A	0.054	*LINC00511*	Huyghe, 2019
rs75954926	17q25.3	G	0.470	*AC144831.1*	Huyghe, 2019
rs4939827	18q21.1	T	0.190	*SMAD7*	Law, 2019
rs285245	19p13.11	T	0.388	*AC020911.2*	Law, 2019
rs10411210	19q13.11	C	0.004	*RHPN2*	Law, 2019

rs1800469	19q13.2	G	0.481	*TMEM91*	Law, 2019
rs12979278	19q13.33	T	0.359	*MAMSTR*	Law, 2019
rs73068325	19q13.43	T	0.033	*MZF1-AS1*	Huyghe, 2019
rs2423279	20p12.3	C	0.230	*AL031679.1*	Law, 2019
rs28488	20p12.3	T	0.250	*BMP2*	Huyghe, 2019
rs6085661	20p12.3	T	0.179	*Intergenic*	Law, 2019
rs961253	20p12.3	A	0.146	*Intergenic*	Law, 2019
rs994308	20p12.3	C	0.156	*Intergenic*	Huyghe, 2019
rs2295444	20q11.22	C	0.035	*PIGU*	Law, 2019
rs2179593	20q13.12	A	0.224	*TOX2*	Law, 2019
rs6065668	20q13.12	C	0.075	*Intergenic*	Law, 2019
rs1810502	20q13.13	C	0.179	*Intergenic*	Law, 2019
rs4811050	20q13.13	A	0.036	*Intergenic*	Law, 2019
rs6066825	20q13.13	A	0.116	*PREX1*	Law, 2019
rs6091213	20q13.13	C	0.074	*Intergenic*	Law, 2019
rs1741640	20q13.33	C	0.389	*LAMA5*	Law, 2019
rs3787089	20q13.33	C	0.427	*RTEL1*	Law, 2019
rs2732875	Xp22.2	C	0.236	*SHROOM2*	Law, 2019
rs5934683	Xp22.2	T	0.496	*GPR143*	Law, 2019

MA, minor allele; MGF, minor genotype frequency

Table S2 Power estimates of different genotype frequencies and effect sizes

	Overall survival		CRC-specific survival	
HR	RGF	Power*	RGF	Power
1.10	0.01	0.001	0.01	0.001
	0.05	0.005	0.05	0.004
	0.10	0.012	0.10	0.009
	0.30	0.064	0.30	0.033
	0.50	0.082	0.50	0.042
1.20	0.01	0.004	0.01	0.003
	0.05	0.055	0.05	0.030
	0.10	0.182	0.10	0.094
	0.30	0.625	0.30	0.384
	0.50	0.696	0.50	0.448
1.30	0.01	0.015	0.01	0.008
	0.05	0.253	0.05	0.131
	0.10	0.639	0.10	0.397
	0.30	0.980	0.30	0.878
	0.50	0.988	0.50	0.908
1.40	0.01	0.041	0.01	0.021
	0.05	0.600	0.05	0.364
	0.10	0.944	0.10	0.779
	0.30	1.000	0.30	0.995
	0.50	1.000	0.50	1.000
1.50	0.01	0.096	0.01	0.048

	0.05	0.878	0.05	0.661
	0.10	0.997	0.10	0.963
	0.30	1.000	0.30	1.000
	0.50	1.000	0.50	1.000
2.00	0.01	0.796	0.01	0.552
	0.05	1.000	0.05	1.000
	0.10	1.000	0.10	1.000
	0.30	1.000	0.30	1.000
	0.50	1.000	0.50	1.000

RGF, rare genotype frequency
*Power was estimated based on the sample size of the discovery cohort (N=5,675) at a type I error rate of 0.0005

Table S3 Effect estimates of rs7495132 and rs10161980 in SOCCS adjusting for age and sex

	OS		CSS	
	HR(95%CI)	p	HR(95%CI)	p
rs7495132	1.40(1.01-1.94)	0.042	1.95(1.40-2.71)	7.80E-05
rs10161980	1.20(1.07-1.35)	0.002	1.19(1.04-1.37)	0.012

OS, overall survival; CSS, CRC-specific survival, HR, hazard ratio; CI, confidence interval;

Table S4 Summarised results of genetic associations (p<0.05 in discovery set) stratified by sex

	**OS**					**CSS**				
	SOCCS			UK Biobank		SOCCS			UK Biobank	
**Male**										
Variant	HR(95%CI)	P(uncorrected)	Pfdr	HR(95%CI)	P	HR(95%CI)	P(uncorrected)	Pfdr	HR(95%CI)	P
rs7495132	1.53(1.01-2.31)	0.045	0.488			**2.35(1.54-3.60)**	**7.976E-05**	**0.010**	1.28(0.56-2.89)	0.560
rs6066825	1.24(1.04-1.46)	0.014	0.488			1.36(1.11-1.65)	0.003	0.166		
rs4811050	1.35(1.01-1.81)	0.041	0.488			1.57(1.15-2.15)	0.005	0.209		
rs9537521	1.22(1.04-1.42)	0.013	0.488			1.23(1.02-1.48)	0.029	0.730		
rs3801081	0.86(0.76-0.96)	0.010	0.488			0.86(0.75-0.99)	0.036	0.730		
rs7993934	0.93(0.83-1.05)	0.224	0.788			0.86(0.75-0.99)	0.038	0.730		
rs3217810	1.51(1.03-2.21)	0.034	0.488			1.59(1.02-2.48)	0.040	0.730		
rs5934683	0.87(0.78-0.98)	0.024	0.488			0.88(0.76-1.01)	0.065	0.903		
rs11893063	0.86(0.74-1.00)	0.043	0.488			0.87(0.73-1.04)	0.126	0.903		
rs11196171	0.72(0.53-0.97)	0.031	0.488			0.78(0.55-1.11)	0.162	0.903		
rs13020391	1.17(1.00-1.38)	0.046	0.488			1.13(0.94-1.37)	0.197	0.903		
rs73975588	0.60(0.37-0.99)	0.044	0.488			0.76(0.44-1.32)	0.329	0.928		
**Female**										
rs174537	**1.48(1.21-1.82)**	**1.639E-04**	**0.021**	0.88(0.66-1.18)	0.398	1.26(0.97-1.63)	0.086	0.659		
rs10161980	**1.38(1.15-1.65)**	**0.001**	**0.041**	1.24(0.98-1.58)	0.076	1.37(1.10-1.69)	0.004	0.542		
rs3217874	1.23(1.04-1.45)	0.014	0.440			1.25(1.03-1.52)	0.024	0.623		
rs9537521	1.29(1.06-1.56)	0.011	0.440			1.21(0.96-1.52)	0.107	0.659		
rs3087967	0.84(0.73-0.97)	0.018	0.450			0.84(0.71-0.99)	0.041	0.659		
rs35509282	0.42(0.20-0.89)	0.023	0.450			0.15(0.04-0.62)	0.008	0.542		
rs4776316	1.34(1.04-1.74)	0.025	0.450			1.17(0.85-1.62)	0.340	0.853		
rs10994860	1.46(1.03-2.06)	0.032	0.456			1.37(0.90-2.08)	0.139	0.659		
rs6065668	0.72(0.54-0.97)	0.030	0.456			0.64(0.44-0.92)	0.016	0.623		
rs3131043	1.20(1.01-1.42)	0.038	0.482			1.21(1.00-1.48)	0.055	0.659		

rs45597035	1.25(1.01-1.55)	0.042	0.491		1.32(1.04-1.69)	0.023	0.623

HR, hazard ratio; CI, confidence interval; Pfdr, p-values adjusted using the false positive rate approach. OS, overall survival, CSS, CRC-specific survival.

Figure S1 Flow chart of patient selection for the two study cohorts

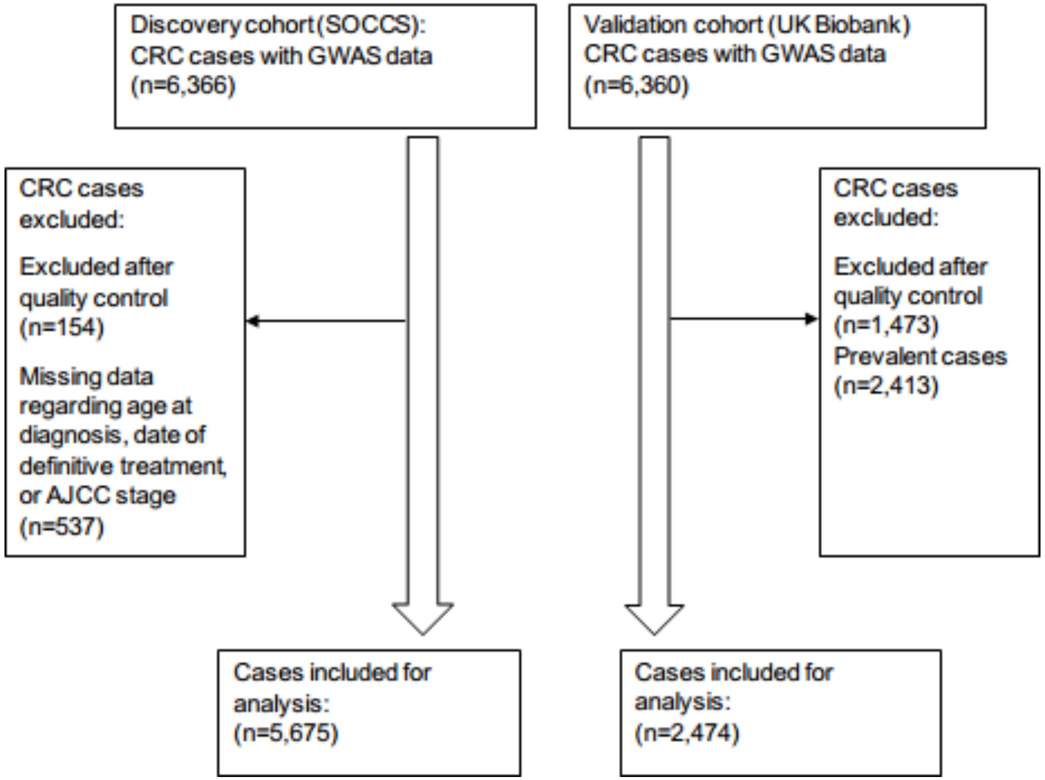

Figure S2 Kaplan-Meier survival estimates of overall survival stratified by rs10161980 and rs7495132

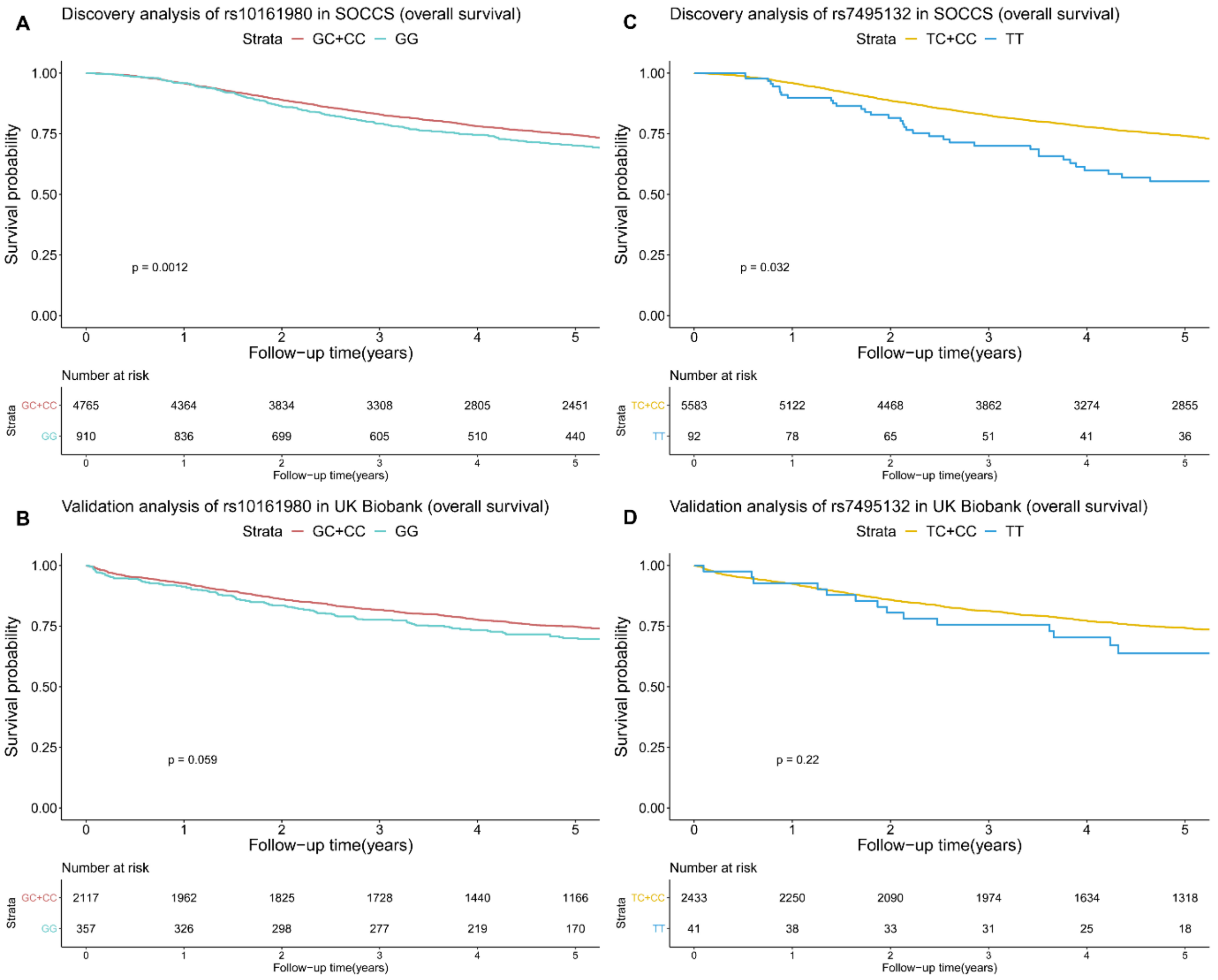

A: rs10161980 in SOCCS, B: rs10161980 in UK Biobank; C: rs7495132 in SOCCS; D: rs7495132 in UK Biobank
